# Application of FDG-PET/CT in Radiation Oncology

**DOI:** 10.3389/fonc.2013.00080

**Published:** 2013-04-11

**Authors:** Jun Li, Ying Xiao

**Affiliations:** ^1^Department of Radiation Oncology, Thomas Jefferson UniversityPhiladelphia, PA, USA

**Keywords:** PET/CT, radiation oncology, radiation therapy, treatment response, treatment planning

## Abstract

Positron emission tomography (PET)/computed tomography (CT), which combines the advantages of high sensitivity and specificity of PET and high resolution of CT, is a unique tool for cancer management. PET/CT has been widely used in cancer diagnosis and treatment. The article reviews the recent applications of PET/CT in radiation oncology, with a focus on ^18^F-fluorodeoxyglucose (FDG)-PET/CT, addressing the applications in treatment planning and treatment response assessment of radiation therapy.

## Introduction

With high sensitivity and specificity, positron emission tomography (PET) is playing an important role in cancer imaging and treatment (Histed et al., 2012; Sveistrup et al., 2012). Combining with computed tomography (CT), PET can provide valuable information on tumor extent for most cancers (Ling et al., 2000). PET/CT has been successfully used in the diagnosis, initial staging, and response assessment in various malignant tumors with high diagnostic accuracy (Borst et al., 2005; Eschmann et al., 2006; Facey et al., 2007) and has been used for PET-guided radiation treatment planning (Jarritt et al., 2006; Gregoire et al., 2007). ^18^F-fluorodeoxyglucose (FDG) is the only Medicare approved PET/CT tracer for cancer imaging and FDG-PET/CT is the most widely available PET/CT procedure used in daily oncology practice. Studies have shown that FDG-PET/CT improves staging accuracy, with a 20–30% improvement in specificity and sensitivity over CT scanning (Toloza et al., 2003; Rodríguez Fernández et al., 2007; Yi et al., 2008). PET/CT systems offer a unique opportunity of improving target localization and facilitating treatment planning for radiation therapy. The advent of integrated or hybrid PET/CT scanners, which has facilitated hardware fusion of PET and CT data sets and improves the accuracy of target localization as compared to the procedures using software fusion of PET and CT scans acquired on separate scanners, have been used popularly nowadays. This study is to review the recent applications of PET/CT in radiation oncology, i.e., in radiation treatment response assessment and treatment planning, with a focus on FDG-PET/CT.

## PET/CT for Radiation Treatment Planning

Positron emission tomography/CT has been increasingly applied for target delineation in radiation treatment planning for a variety of cancer treatments, e.g., cervix, lung, head and neck (HN), and prostate, etc. (Erdi et al., 2002; Bradley et al., 2004; Lavrenkov et al., 2005; Nestle et al., 2005; Paulino et al., 2005; Greco et al., 2007; Lin et al., 2007; van Baardwijk et al., 2007; Dolezelova et al., 2008; Henriques de Figueiredo et al., 2009; MacManus et al., 2009; Yu et al., 2009; De Jong et al., 2010; Terezakis et al., 2011; Lee et al., 2012).

Studies showed that FDG-PET/CT improved the accuracy of target definition (Nestle et al., 2006; MacManus et al., 2009) and PET/CT reduced the inter-observer variability compared to CT alone (Tejwani et al., 2012). PET/CT helps in finding CT-undetected or borderline sized nodes and improves target accuracy for nodal radiation. For lung cancer, PET/CT showed high sensitivity and specificity for mediastinal lymph node involvement over CT. FDG-PET/CT can be used to differentiate tumor from collapsed lung and normal tissue, and defines disease extent in the chest wall. Studies had showed the differences of volumes contoured based on PET/CT and those contoured based on CT alone. For HN cancer, PET/CT can identify metastatic nodal disease which CT cannot. Studies have showed significant differences between PET/CT-derived and CT-derived tumor volumes in HN patients. Garg et al.’s (2012) study showed that PET/CT led to modification in treatment planning in 55% of the HN patients studied. For cervical cancer, PET/CT has showed high sensitivity and specificity in initial staging and restaging cervical cancer and PET/CT has the advantage of detecting gross para-aortic and pelvic lymph nodes (PLNs) for treatment planning, which CT may not be able to detect. Incorporation of PET/CT into radiotherapy planning has the potential to allow radiation-dose escalation without increasing side effects (De Ruysscher et al., 2005; Pinkawa et al., 2012). Radiation treatment can be improved by using PET/CT for target volume delineation: doses to the tumor can be increased and organs at risk (OARs) can be spared. A summary of PET/CT applications in radiation treatment planning is provided in Table 1.

**Table 1:** **Application of PET/CT in radiation treatment planning**.

Reference	Site of tumor	Results/Conclusion
Dolezelova et al. (2008)	Cervix (external beam and HDR)	PET/CT plays an important role in diagnosis and treatment of cervical carcinoma and in determination of target volumes
Lin et al. (2007)	Cervix (LDR and HDR)	FDG-PET/CT-based treatment planning allowed for improved dose coverage of the tumor without significantly increasing the dose to the bladder and rectum
Tejwani et al. (2012)	Cervix	Inter-observer GTV variability decreased in PET/CT-based planning compared to CT-based planning
Paulino et al. (2005)	Head and neck	PET/CT-based GTVs were different from CT-based GTVs in most cases
Henriques de Figueiredo et al. (2009)	Head and neck	Volume comparison showed a reduction and qualitative discrepancies between the PET- and CT-volumes
Garg et al. (2012)	Head and neck	PET/CT led to a modification in treatment planning in 55% of patients studied
De Jong et al. (2010)	Prostate	Review of PET/CT and radiotherapy in prostate cancer patients
Pinkawa et al. (2012)	Prostate	Treatment planning with (18)F-choline PET-CT allows a dose escalation to a macroscopic intraprostatic lesion without significantly increasing toxicity
Terezakis et al. (2011)	Lymphoma	PET/CT-based treatment planning for lymphoma patients resulted in considerable changes in management, volume definition, and normal tissue dosimetry
Yeoh and Mikhaeel (2013)	Lymphoma	Critical review of incorporating PET/CT into radiation therapy of lymphoma
Erdi et al. (2002)	NSCLC	There was a change in PTV outline based on CT images versus CT/PET fused images
Bradley et al. (2004)	NSCLC	Biologic targeting with PET alters the radiation treatment volume significantly in 30–60% of NSCLC patients for whom definitive therapy is planned
Greco et al. (2007)	NSCLC	Significant impact of PET-derived contours on treatment planning was shown in 30–60% of the plans with respect to the CT-only target volume
van Baardwijk et al. (2007)	NSCLC	Source-to-background ratio-based auto-delineation showed a good correlation with pathology, decreased the delineated volumes of the GTVs, and reduced the inter-observer variability
Nestle et al. (2005)	NSCLC	Different techniques of tumor contour definition by (18)F-FDG-PET in radiotherapy planning lead to substantially different volumes
Yu et al. (2009)	NSCLC	Integrated 18F-FDG-PET/CT is an effective tool to define the target of GTV in radiotherapy
Lavrenkov et al. (2005)	NSCLC	PET results in a reduction in the CT-derived GTV for NSCLC primary target volume in 15% of the patients
De Ruysscher et al. (2005)	NSCLC	The use of a combined dedicated PET/CT allowed significant radiation-dose escalation whilst respecting all relevant normal tissue constraints
Nestle et al. (2006)	NSCLC	Review of technical factors influencing PET and PET/CT data, and their consequences for radiotherapy planning
Lee et al. (2012)	Lung	Review of FDG-PET/CT-based radiation treatment planning for lung cancer

*Treatments using brachytherapy are indicated*.

Positron emission tomography/CT images are used in two ways in radiation treatment planning: for PET/CT images acquired from a diagnostic scanner, the images are registered/fused with planning CT images; for PET/CT images acquired on a dedicated planning PET/CT scanner, the images are directly used for treatment planning. To register/fuse the diagnostic PET/CT images acquired in non-treatment position, with planning CT images acquired in treatment position, software for image registration/fusion are needed. Usually rigid image registration is performed. Rigid image registration accounts for only linear or uniform transformation within six degrees of freedom. Recently, deformable registration which can account for significant temporal and anatomic changes between the image sets, has been applied for PET/CT-CT image fusion. The study of Kovalchuk et al. (2012) has demonstrated that deformable registration is a powerful tool for the image fusion of diagnostic PET/CT and planning CT for target volume delineation.

The use of dedicated PET/CT systems for treatment planning is increasing in radiation oncology. The advantage is that the system produces co-registered images with the patient in the treatment position and the images can be used directly for treatment planning without image fusion with another planning CT. Thus the accuracy of target volume delineation is improved.

For target volume delineation on PET/CT images, manual or automated method is used. It is challenging to identify lesion edges in noisy PET data. The manual delineation relies on clinician’s expertise and is also limited by image display settings, e.g., window level and width. The automated delineation is based on quantitative or semi-quantitative techniques derived from the standardized uptake value (SUV), which assesses the level of FDG uptake. The basic idea is to decide a cutoff of measured SUV to separate target from background tissues. Various techniques of automated delineation have been studied (Lee, 2010). There are concerns of the accuracy of automated delineation. Yeoh and Mikhaeel’s (2013) paper emphasized that one must be cautious when adopting automated volume delineation using PET/CT information because there can be significant variation depending on the parameters and segmentation techniques used.

A limiting factor for accurate target volume delineation by PET/CT is organ and tumor motion caused by patient respiration. Respiration introduces artifacts in CT and PET images, which can result in degraded image quality and can lead to possible tumor missing from treatment volumes or under-treatment. Methods have been developed for motion management in PET/CT for radiation treatment planning, which include 4D PET/CT and deep inspiration breath-holding PET/CT (Nehmeh et al., 2004, 2007; Bettinardi et al., 2012; Scripes and Yaparpalvi, 2012).

## PET/CT for Treatment Response Assessment

^18^F-fluorodeoxyglucose-PET/CT has been frequently used to monitor the response of cancer treatment (Rege et al., 2000; Choi et al., 2002; Jeong et al., 2002; Hicks et al., 2004; Hicks, 2005; Gagel et al., 2006; Juweid and Cheson, 2006; Pöttgen et al., 2006; Bussink et al., 2011; Porceddu et al., 2011; Caldarella et al., 2012; Cheebsumon et al., 2012; Huh et al., 2012; Janssen et al., 2012; Perez et al., 2012; Vaidya et al., 2012; Lee et al., 2013). Studies have demonstrated the potential of using FDG to predict response and survival in different cancer sites (Grigsby et al., 2004; Hicks et al., 2004; Kalff et al., 2006; Petit et al., 2009; van Loon et al., 2011; van Stiphout et al., 2011). Kidd et al. (2010) evaluated the prognostic significance of the maximum SUV(max) of FDG in a study of 83 cervical cancer patients. In the study, the SUV(PLN) was analyzed for its association with treatment response, pelvic disease recurrence, disease-specific survival, and overall survival. The SUV(PLN) was found to be correlated with an increased risk of persistent disease after treatment (*P* = 0.0025), specifically within the PLN region (*P* = 0.0003), and was found to be predictive of an increased risk of ever developing pelvic disease recurrence (*P* = 0.0035). Patients with a higher SUV(PLN) were found to have significantly worse disease-specific (*P* = 0.0230) and overall survival (*P* = 0.0378). The study showed that SUV(PLN) is a prognostic biomarker, which can predict treatment response, pelvic recurrence risk, and disease-specific survival in patients with cervical cancer. A summary of PET/CT applications in radiation treatment response is provided in Table 2.

**Table 2:** **Application of PET/CT in radiation treatment response**.

Reference	Site of tumor	Results/Conclusion
Bussink et al. (2011)	Various	(Review) Discussion of the potential of integrated PET-CT for treatment selection, response monitoring early after the start of treatment, and prediction of outcome for solid tumors
Caldarella et al. (2012)	Osteosarcoma	Review of FDG-PET/CT in assessing response to neoadjuvant treatment in patients with osteosarcoma
Cheebsumon et al. (2012)	NSCLC	PET-based tumor delineation methods provided tumor sizes in agreement with pathology
Choi et al. (2002)	NSCLC	Correlation between the gradient of residual metabolic rate of glucose after chemoradiotherapy and the probability of tumor control on the basis of pathologic tumor response is an inverse dose-response relationship
Grigsby et al. (2004)	Cervix	Post-therapy abnormal FDG uptake (persistent or new) as detected by whole-body PET measures tumor response and might be predictive of tumor recurrence and death from cervical cancer
Hicks et al. (2004)	NSCLC	Post-radiotherapy inflammatory changes detected by FDG-PET are positively correlated with tumor response
Hicks (2005)	Various	(Review) The potential benefits and limitations of FDG-PET were discussed
Huh et al. (2012)	Rectum	The FDG-PET/CT parameters and the response index may be best for assessing the neoadjuvant chemoradiation response of locally advanced rectal cancer
Janssen et al. (2012)	Rectum	The presented predictive model could be used to select patients to be considered for less invasive surgical interventions or even a “wait and see” policy
Jeong et al. (2002)	NSCLC	(18)F-FDG uptake correlated with survival in NSCLC
Juweid and Cheson (2006)	Various	(Review) The use of 18F-FDG-PET in the assessment of cancer after therapy, including restaging tumors and monitoring tumor response, was discussed
Kalff et al. (2006)	Rectum	Post-chemoradiation (18)F-FDG-PET scintigraphy provides good medium-term prognostic information in patients with advanced rectal cancer undergoing radical surgery with curative intent
Kidd et al. (2010)	Cervix	SUV is a prognostic biomarker, predicting treatment response, pelvic recurrence risk, and disease-specific survival
Lee et al. (2013)	Cervix	Significant decreases in tumor volume were observed on PET/CT images during and after concurrent chemoradiotherapy
Perez et al. (2012)	Rectum	Assessment of tumor response at 12 weeks after chemoradiation completion with PET/CT imaging may provide a useful additional tool with good overall accuracy for the selection of patients
Petit et al. (2009)	NSCLC	A methodology was presented to derive relationships between FDG uptake, dose, and metabolic control
Porceddu et al. (2011)	Head and neck	PET-directed management of the neck after definitive RT in node-positive HNSCC appropriately spares neck dissections in patients with PET-negative residual CT nodal abnormalities
Pöttgen et al. (2006)	NSCLC	SUV values from two serial PET/CT scans, before and after three chemotherapy cycles or later, allow prediction of histopathologic response in the primary tumor and mediastinal lymph nodes and have prognostic value
Rege et al. (2000)	Head and neck	Pretreatment PET findings may have prognostic implications in determining which patients will achieve long-term local control with primary radiation therapy
Vaidya et al. (2012)	NSCLC	Multimodality image-feature modeling provides better performance compared to existing metrics and holds promise for individualizing radiotherapy planning
van Loon et al. (2011)	SCLC	Both early CT and FDG-metabolic tumor volume changes show a significant correlation with survival in SCLC
van Stiphout et al. (2011)	Rectum	The model and the nomogram developed based on clinical and sequential PET-CT data can accurately predict pathologic complete response

A study has demonstrated that a high SUV for FDG in the primary tumor and regional nodes after completion of radiotherapy predicted poor treatment response and tumor control in non-small cell lung cancer (Jeong et al., 2002). Rege et al.’s (2000) study in HN patients showed that PET findings might have prognostic implications in determining which patients will achieve long-term local control with primary radiation therapy and might help identify those patients at increased risk of recurrence that may benefit from more aggressive altered fractionation schemes or combined modality therapy. Jeong et al.’s (2002) study showed that the detection of residual and recurrent disease by FDG-PET/CT has a reported sensitivity of 100%, specificity of 92%, positive predictive value of 92%, negative predictive value of 100%, and diagnostic accuracy of 96%.

A recent study enrolling 50 patients with locally advanced rectal cancer assessed the value of sequential FDG-PET/CT scans for predicting the response of locally advanced rectal cancer to neoadjuvant chemoradiation (Huh et al., 2012). The treatment consisted of concurrent chemoradiation, which included preoperative 5-fluorouracil-based chemotherapy and pelvic radiation (4500 to 5040 cGy). All the patients underwent FDG-PET/CT before and 5 weeks later (median: 35 d) after the completion of chemoradiation. After chemoradiation, 32 of 50 patients (64%) were classified as responders according to the tumor regression grade. For all the patients, the mean pre-chemoradiation SUV(max) was significantly higher than the mean SUV(max) value at post-chemoradiation (*P* < 0.001). The mean response index was significantly higher in the responders than that in the non-responder patients (*P* = 0.001). The study concluded that the FDG-PET/CT parameters and especially the mean response index, may be best for assessing the neoadjuvant chemoradiation response of locally advanced rectal cancer and those values can potentially assist physicians for planning the optimal treatment.

## Conclusion

Positron emission tomography/CT is being actively used in radiation oncology for treatment response assessment and treatment planning. Careful attention needs to be paid to the details in applications, e.g., image fusion, automated image segmentation, and patient motion management. PET/CT, a valuable tool for radiation oncology, is bringing significant impact on radiation treatment.

## Conflict of Interest Statement

The authors declare that the research was conducted in the absence of any commercial or financial relationships that could be construed as a potential conflict of interest.
